# How to select patients requiring coronary revascularisation using coronary physiology

**DOI:** 10.1177/2048004020979476

**Published:** 2021-02-03

**Authors:** Bhavik Modi, Divaka Perera

**Affiliations:** NIHR Biomedical Research Centre and British Heart Foundation Centre of Excellence, School of Cardiovascular Medicine and Sciences, St Thomas’ Campus, King’s College London, London, UK

**Keywords:** Catheter-based coronary interventions, stents, chronic ischemic heart disease, acute coronary syndromes

## Abstract

The coronary angiogram is an indicator of flow limiting coronary artery disease but coronary physiology at the time of angiography is vital in assessing the true functional significance of coronary artery disease. With advances in guidewire technology and the greater use of physiology within the catheter laboratory, there is now a slow evolution of physiological indices in being able to reliably assess the functional significance of individual lesions and also the adequacy of revascularization in a growing range of clinical scenarios. As co-registration of physiology with the angiogram and intravascular imaging will become easier, we will find ourselves increasingly in an era of ‘Precision PCI’.

## Introduction

**Figure 1.**
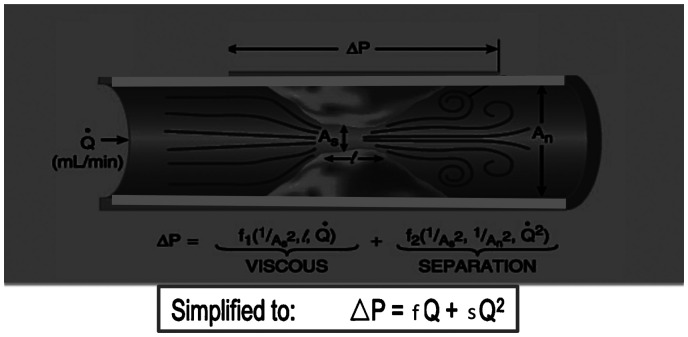
Pressure gradients across a stenosis can be described by a quadratic relationship between pressure and flow that involve the energy losses due to the flow separation within a vessel (s) and the unique frictional coefficient (f) of a stenosis. This takes the form of ΔP = fQ + sQ, in which ΔP indicates change in pressure, f indicates the frictional coefficient of a lesion (influenced by lesion length and diameter stenosis), s indicates the separation coefficient of flow conditions in a vessel, and Q indicates flow.

The angiogram has been considered the gold standard in detecting coronary artery disease for the last five decades. The underlying principle is lumenography, which has several limitations, not least the fact that it is a 2-dimensional representation of a 3-dimensional structure with the same stenosis appearing markedly different in different planes. Furthermore, identifying the severity of a narrowing on angiography relies on identifying an accurate vessel reference diameter, which is prone to error especially when there is diffuse atheroma throughout a vessel. Although newer techniques have been developed to assess vessel anatomy in greater detail, such as intravascular ultrasound (IVUS) and Optical Coherence Tomography (OCT), inferring the functional impact of a stenosis based on its structure is complex.^1^ Several factors influence the haemodynamic changes within a diseased coronary artery, not just the stenosis diameter. These include the length of a stenosis, stenosis geometry and flow velocity within the vessel; which in turn depends on the state of the microvasculature and the amount of myocardium subtended by the stenotic vessel in question (Figure 1).^2^ In this article, we review the techniques available for assessing coronary physiology at the time of angiography and the practical applications as well as inherent limitations of each.

## Fractional flow reserve

The pressure gradient across a stenosis (ratio of distal coronary to aortic pressure, Pd/Pa) during maximal adenosine-induced hyperaemia is referred to as Fractional Flow Reserve (FFR) and is the most widely used marker of the functional significance of coronary artery disease. During conditions of hyperaemia, coronary autoregulation is temporarily disabled and flow in the distal vessel is assumed proportional to the change in pressure across it.

FFR was initially validated nearly three decades ago, against non-invasive surrogate markers of ischaemia in the setting of stable angina.^3,4^ These early studies suggested that a binary FFR threshold of 0.75 accurately identifies patients who have an abnormal non-invasive ischaemia test and that it is safe to defer intervention on vessels associated with a FFR value higher than this threshold. It has since also been shown that the visual assessment of angiographic stenosis severity does not reliably correlate with physiological assessment , leading to a misclassification of stenoses in around a third of lesions.^5,6^ Perhaps as a consequence, a body of evidence has accumulated that FFR-guided revascularisation confers significant clinical and prognostic benefit over management based on angiography alone, particularly for stable angina,^7–10^ and potentially managing non-culprit vessels in NSTEMI settings.^11^

## Resting physiological indices

**Figure 2.**
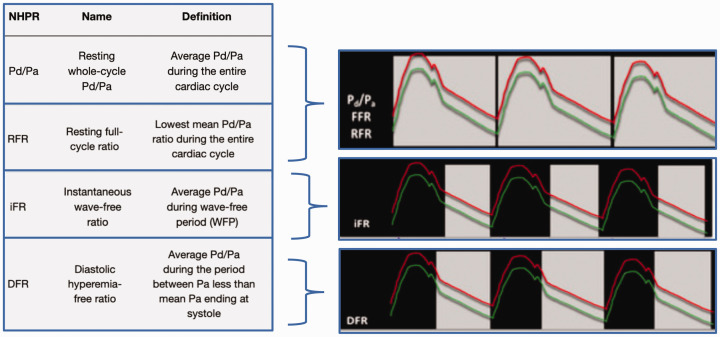
Four commonly used Non-Hyperaemic Pressures Ratios (NHPR): Resting Pd/Pa, RFR, iFR and DFR. The Red line represents the aortic pressure, the green line the distal coronary pressure and the gray shaded area represents the measurement period.

Despite the evidence for physiology guided management of coronary disease, the uptake has been limited, with many patients being put forward for percutaneous or surgical revascularisation based on angiographic appearances alone. While there are many behavioural and fiscal explanations for this, one potential factor is the perceived complexity of physiological assessment in the cardiac catheter laboratory. In view of this, there has been growing interest in techniques that are based on resting pressure measurement alone, without the need for induction of hyperaemia (collectively referred to as non-hyperemic pressure ratios, NHPR): summarised in Figure 2.

Currently, the most widely used NHPR is the instantaneous wave-free ratio (iFR). iFR is defined as P_d_/P_a_ ratio during the latter 75% of diastole (minus the last 5ms), when resistance is purported to be constant and pressure can be assumed proportional to flow, without the need to modulate resistance with adenosine-induced hyperaemia. iFR-guided management has recently been shown to have comparable investigator determined revascularisation rates to FFR, in a relatively low-risk group of patients, with data awaited on more complex subsets.^12,13^ The design and conclusion of these studies has drawn some criticism. The data for iFR is driven by the DEFINE-FLAIR and iFR-SWEDE-HEART trials, which were non-inferiority trials with a non-inferiority margin that has been suggested to be generous. A pooled meta-analysis subsequently suggested a numeric excess of death and MI events occurred in the iFR group (albeit statistically non-significant (p = 0.09).^14^ In addition, there is growing evidence to suggest the wave-free period is not associated with a fixed minimal microvascular resistance with NHPR indices showing greater test-retest variability.^15^ Furthermore, there now good evidence that there is actually no significant difference between iFR and other NHPR indices, such a resting Pd/Pa or Resting Flow Ratio (RFR), both numerically and with respect to their agreement with FFR.^16^ In our opinion, NHPR indices are similar to each other, all share the relative ease afforded by not needing to induce hyperaemia but by the same token, are similarly limited in accuracy.

## Threshold creep

Mounting evidence suggests that patients on optimal medical therapy alone have an excellent prognosis, as suggested by the COURAGE trial^17^ and more recently the ISCHEMIA trial. On the other hand, physiology-guided revascularization, is associated with improved outcomes, with data now existing out to 5 years from the original FAME studies.^6^ Data also exists to suggest patients with a lower FFR are most likely to derive prognostic and symptomatic benefit.^18^

The initial derivation studies of FFR were performed against a combination of non-invasive tests and showed an optimum threshold of 0.75 to identify ischaemia.^4^ The first randomized trial to show improved outcomes with revascularization guided by FFR also used a threshold of 0.75.^5^ Subsequent RCT’s to investigating whether there are improved clinical outcomes with FFR guided revascularisation were done using the higher threshold of 0.80 in the FAME trials (giving the “safety net” of the greater negative predictive value at the 0.80 threshold).^7,19^ Following this, iFR was also originally derived and validated against an FFR ≤ 0.80 threshold with the ADVISE^20^ and CLARIFY^21^ studies suggested that iFR thresholds of 0.83 and 0.86 provided optimal agreement with the FFR threshold of 0.8. Despite this, again a “safety net” of a higher 0.89 threshold was used for the iFR-Swedeheart and DEFINE-Flair trials.

This ‘upward creep’ of contemporary thresholds may have resulted in suboptimal diagnostic accuracy compared to an FFR threshold of 0.75 and iFR threshold of 0.86 (cut-offs in original derivation studies).^22^ Whether patients would derive greater benefit if physiology guided revascularisation was guided by the more stringent original thresholds remains to be seen.

## Physiology in special scenarios: LMCA disease

Whilst evidence supporting physiology-guided revascularization has grown in recent years, most of the trials such as DEFER and the FAME have excluded LMCA disease.^23^ This is despite the fact that the LMCA is even more prone to angiographic error compared to the vessels to angulation and overlap.^24^ There are some observational data that suggests FFR guided LMCA revascularization is safe but the individual studies were small, incorporated different thresholds and different modes of revascularization.^24,25^

**Figure 3.**
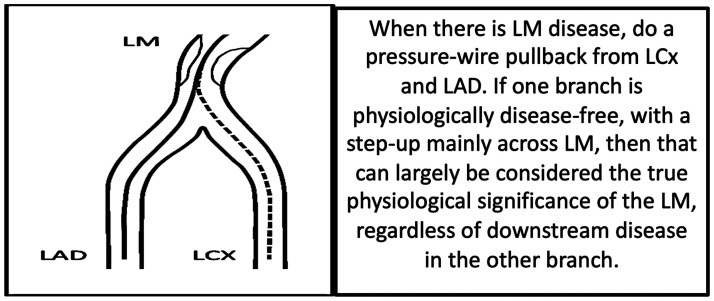
A method of assessing the Left Main (LM) physiologically when there is a disease-free daughter vessel, either there Left Anterior Descending (LAD) or Left Circumflex (LCx).

Practically assessing the functional significance of LMCA disease is slightly more complex than downstream single vessel disease. Firstly, there are technical challenges with the need to disengage the guide catheter and equalise in the aortic root. This also means there is a need for intravenous adenosine as opposed to intracoronary adenosine. Once FFR is measured, there is the issue of serial coronary artery disease with downstream disease in the LAD and/or LCx found in the majority of cases. Numerous data suggest significant error (usually in the form of underestimation) in identifying the true stenosis significance in the presence of serial downstream coronary artery disease. As a result, it is recommended that when a LMCA stenosis is assessed physiologically, this should be done with guidance from a pressure wire pullback from both the LCx and LAD^23^ (Figure 3).

When a left main stenosis is found in the presence of a physiologically disease-free side branch, there is evidence from animal studies, with human validation, to suggest the true LMCA stenosis can be isolated by measuring into the disease-free vessel (Figure 3). Whilst small errors are still possible with this method, the error is only most marked when the FFR in the serially diseased vessel is <0.45.^26^ When there is downstream disease in both vessels, physiological assessment of true LMCA significance becomes more of a challenge. In the absence of a validated and robust technique, the practice of treating the greatest FFR step-up following manual FFR pullback appears to be the best available method for LMCA physiology-guided PCI and has been shown to be safe and reduce unnecessary revascularisation with acceptable outcomes at a mean follow-up period of 9 months.^27^ IVUS can also be useful in helping assess LMCA significance, with IVUS thresholds in the region of 4.5–7.5 mm^2^ providing some improvement to angiography alone in determining the functional significance of a LMCA stenosis.^28–30^

## Physiology in special scenarios: Serial disease

The data supporting physiology-guided revascularisation are largely derived from studies that only included vessels with single discrete lesions. The reality, particularly within elderly and diabetic populations, is that serial and diffuse CAD is common.^31^ As discussed in the section on physiological assessment of LMCA disease, it is unclear if physiological indices can reliably assess individual lesions in the commonly-encountered scenario of serial/diffuse coronary artery disease, with significant stenosis underestimation having been shown, even when relying on a pressure wire pullback method^32^ (Figure 2).

It has been purported that resting physiological indices, such as iFR, are associated with less serial stenosis interaction but data now exists to suggest all pressure-derived indices, resting and hyperaemic, are prone to significant error and stenosis misclassification, perhaps with resting indices only marginally less so.^33^ To correct for some of this error, a simple equation to use with hyperemic pressure wire pullback has been derived with a 3-D printed model and validated in patients with serial disease to show a significant reduction error and stenosis misclassification.^32,33^

Until novel FFR/iFR correction equations are studied further, we recommend making decisions based on the size of the step-up observed during hyperarmic or resting pressure wire pullback manoeuvres, both before and after PCI. The pullback should be done at a steady speed from the distal vessel, without stopping between stenoses, and should be performed even when a stenosis appears angiographically discrete, as there maybe be accompanying diffuse disease in the vessel that would affect the pressure gradient across a given stenosis. The pullback should be done with knowledge that the accompanying disease is most commonly underestimated, and the extent of this underestimation is greater when there is a large cumulative pressure drop in the vessel.^32^

## Physiology in special scenarios: Acute coronary syndromes

FFR has largely been validated for use in the stable coronary disease and its applicability in the acute setting is less clear. In the case of ST-elevation myocardial infarction (STEMI), impaired microcirculation in the infarction region has been shown to influence FFR measurements in the culprit vessel for up to 6 months after the event.^34^ However, FFR has been used to guide revascularisation in non-culprit vessels with some data to suggest a better outcome than angiographically-guided revascularisation, although definitive data is awaited on this with ongoing trials.^35^

In the NSTEMI setting, in the absence of clear microcirculatory impairment and heavy thrombus load, FFR measurements performed during angiography can be useful to guide revascularization.^36^ Decisions about deferral of revascularisation, however, might need to be made more cautiously for these patients with some evidence to suggest higher FFR thresholds may be safer for deferral in the ACS setting.^37^

## Physiology in other special scenarios

### Vein grafts

There are limited data on FFR measurements in stenotic bypass vessels. A non-randomised study has shown that an FFR-guided PCI strategy of intermediate bypass graft stenoses resulted in lower MACE rates compared to an angiographic-guided strategy,^38^ but this study is hypothesis generating only and these patients were excluded from the major trials, such as FAME.^7,8^ In summary, FFR assessment of bypass grafts should be done with caution and stenoses of downstream native vessels should be considered native serial stenoses, with the same caveats applicable.

### Chronic total occlusions

When a donor vessel supplies a chronic total coronary occlusion (CTO), the FFR and iFR in that vessel should be interpreted with caution as it takes into account the total burden of disease (not just in the donor vessel, but also the viable territory supplied by the CTO vessel). This is supported by data to show that upon successful PCI of the CTO, the donor vessel FFR and iFR show an immediate and sustained increase.^39,40^

### Aortic stenosis

Patients with significant aortic stenosis were excluded from the major coronary physiology trials such as FAME and FAME-2.^7,8^ The reliability of physiological indices is still not established for severe Aortic Stenosis, despite recent data shedding light on the haemodynamic implications of severe aortic stenosis.^41,42^ Overall, recent data demonstrate that FFR-guided revascularization is feasible and safe, and results in deferral of unnecessary stenting in a large proportion of patients with Aortic Stenosis,^43^ but FFR should be interpreted cautiously in this group of patients with heterogenous responses to adenosine and variable microvascular dysfunction.

### LV dysfunction

Reduced Ejection Fraction has been shown in sub-group analyses of the FAME trial to have no significant influence on FFR, unless the stenosis is very tight (FFR < 0.6), in which case clinical overestimation might occur.^44^ This subset of patients does however often have elevated right atrial pressures. Right atrial pressure measurement originally formed part of the FFR calculation, but for the purpose of simplicity, is assumed to be zero. It has however been shown in a small cohort study that ignoring venous pressures (or assuming an arbitrary fixed value such as 5 or 10 mmHg) results in significant errors in FFR measurement, especially at lower values of P_d_ and hence FFR.^45^ While FFR-guided revascularisation has been shown to be valid in patients with LV dysfunction, this is another sub-group where it should be interpreted cautiously.

## Assessing the adequacy of revascularisation with physiology

Coronary physiology is used even less by operators to detect the adequacy of revascularisation. This is despite the fact that we have known for a while that low FFR after PCI in an angiographically optimised vessel portends a worsened prognosis.^46^ Sub-optimal Post-PCI FFR and iFR have both been subsequently shown to be associated with worse outcomes, although recent data suggests that compared with post-PCI FFR, post-PCI Pd/Pa shows limited reclassification ability for the occurrence of TVF.^47^

The DEFINE-PCI study showed that post-PCI physiological assessment detected residual ischemia in nearly 1 in 4 patients after coronary stenting despite an operator-determined angiographically successful result,^48^ with recently presented 1-year data from this study suggesting patients with residual ischaemia were more symptomatic (TCT Connect 2020, yet to be published). Data from the TARGET-FFR study was also recently presented at TCT Connect 2020, which showed that sub-optimal results after angiography-guided PCI are common and checking physiology post PCI results in fewer patients leaving the catheter laboratory with an FFR<0.80.

## Future directions in coronary physiology

We believe the next 10 years will see coronary physiology evolve in several ways. Firstly, despite the evidence to support physiology guided revascularisation, the perceived ‘extra-step’ often prevents clinicians from using physiology, particularly in developing countries. There is now an increased effort from manufacturers to develop pressure wires that perform equivalently to standard workhorse guidewires. An example is the COMET II™ from Boston Scientific which has been developed with Asahi to encourage angioplasty on the same wire after physiological assessment.

**Figure 4.**
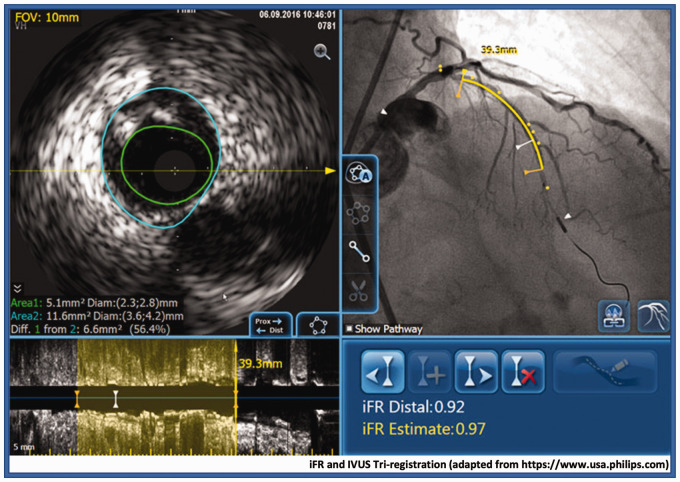
Philips developed ‘iFR and IVUS tri-registration’ that is enables IVUS and iFR pullback data to be co-registered onto the angiogram. The yellow line marries up with the IVUS section of interest and the yellow dots represent a 0.01 iFR unit of step up. Adapted from www.usa.philips.com.

Another advance that is being made is the improvements in co-registration of physiology in the catheter laboratory. As well as co-registration of physiology with the angiogram, there is an increasing drive to co-register physiological data with intravascular imaging. An exciting co-registration tool currently in development is Optical Coherence Tomography (OCT) derived FFR (so called ‘OFR’) – this potentially has the advantage of being able to do physiological and anatomical assessment of a vessel on a standard workhorse guidewire. Data for the latest iteration of OFR suggests it substantially improved the accuracy in identifying hemodynamically significant lesions compared with OCT alone. The study reported that the area under the curve was 0.93 for OFR and 0.80 for OCT-derived MLA.^49^ Whilst this is a developing tool with further data awaited, what we have currently available to clinicians is Philips developed ‘iFR and IVUS tri-registration’ that enables IVUS and iFR pullback data to be co-registered onto the angiogram (see Figure 4). Whilst Philips and iFR have led the way, other physiological and intravascular imaging tools will catch up over the next decade with an era of *‘Precision PCI’* awaiting us.

In addition to guidewire and co-registration advances, there is also a move towards being able to diagnose microvascular coronary artery disease in the catheter laboratory as easily as diagnosing flow-limiting epicardial coronary artery disease, with potential therapies being studied.^50,51^ With the use of thermodilution boluses on a standard pressure wire, clinicians can measure the Index of Microvascular Resistance (IMR = Pd × mean transit time of room temperature saline during maximal hyperaemia) and Coronary Flow Reserve (CFR = coronary flow in hyperaemia/coronary flow at rest). An in-depth discussion of advances in microvascular coronary physiology is beyond the scope of this review but is certainly a growing field and can no longer be ignored by clinicians.

## Conclusions

The coronary angiogram is a useful indicator of flow limiting coronary artery disease but coronary physiology at the time of angiography is vital in assessing the true functional significance of coronary artery disease. With the greater use of physiology within the catheter laboratory, there is now a slow evolution of physiological indices in being able to reliably assess the functional significance of individual lesions and also the adequacy of revascularization in a growing range of clinical scenarios.

As we move towards an era of precision PCI, co-registration of physiology with the angiogram and intravascular imaging will become common and with improvements in pressure-wire technology, clinicians will find it easier to physiologically optimise their PCI outcomes.
